# MicroRNAs in the DNA Damage/Repair Network and Cancer

**DOI:** 10.1155/2014/820248

**Published:** 2014-01-30

**Authors:** Alessandra Tessitore, Germana Cicciarelli, Filippo Del Vecchio, Agata Gaggiano, Daniela Verzella, Mariafausta Fischietti, Davide Vecchiotti, Daria Capece, Francesca Zazzeroni, Edoardo Alesse

**Affiliations:** Department of Biotechnological and Applied Clinical Sciences, University of L'Aquila, Via Vetoio, Coppito 2, 67100 L'Aquila, Italy

## Abstract

Cancer is a multistep process characterized by various and different genetic lesions which cause the transformation of normal cells into tumor cells. To preserve the genomic integrity, eukaryotic cells need a complex DNA damage/repair response network of signaling pathways, involving many proteins, able to induce cell cycle arrest, apoptosis, or DNA repair. Chemotherapy and/or radiation therapy are the most commonly used therapeutic approaches to manage cancer and act mainly through the induction of DNA damage. Impairment in the DNA repair proteins, which physiologically protect cells from persistent DNA injury, can affect the efficacy of cancer therapies. Recently, increasing evidence has suggested that microRNAs take actively part in the regulation of the DNA damage/repair network. MicroRNAs are endogenous short noncoding molecules able to regulate gene expression at the post-transcriptional level. Due to their activity, microRNAs play a role in many fundamental physiological and pathological processes. In this review we report and discuss the role of microRNAs in the DNA damage/repair and cancer.

## 1. Introduction

The DNA damage repair (DDR) response is an intricate signal transduction pathway activated upon DNA damage. To preserve the genomic integrity, due to various endogenous and exogenous stimuli (i.e., UV, ionizing radiations IR, reactive oxygen species ROS, and genotoxic drugs), cells activate specific signaling networks to arrest cell cycle progression, enabling the damage repair, or to proceed toward apoptosis, when the DNA lesions are too severe and not retrievable [1]. Many genes involved in these processes have been studied and characterized at the transcriptional and post-translational level. In the last decade, microRNAs, a new class of molecules able to post-transcriptionally regulate gene expression, have emerged to be involved in several fundamental physiological and pathological biomolecular and cellular mechanisms. Cancer cells often show significant alterations at the level of the DDR response and develop resistance to DNA damage-inducing agents. In this review, we illustrate the involvement of miRNAs in regulatory networks affecting the DNA damage/repair process in several types of solid tumors.

## 2. The DNA Damage Response: An Overview

The DDR is a kinase-based functional network primarily activated in response to stalled replication forks, incomplete DNA replication, and different types of DNA lesions. It initiates signaling cascades to activate cell cycle checkpoints [1]. The DDR is triggered by early phosphorylation-driven signaling cascades followed by a delayed response that acts at the transcriptional level and promotes the induction of Cdk inhibitors, extending the time of cell cycle arrest [2, 3]. Early signaling events include the activation of ATM, ATR, and DNA-PKc, the phosphorylation of H2AX, and the recruitment of the complexes Mre11-Rad50-Nbs1 or Rad9-Hus1-Rad1 at the level of damaged sites [4]. The ATM kinase initiates a signaling pathway mainly induced by DNA double-strand breaks (DSBs) and acts by phosphorylating hundreds of proteins [5]. Chk2 is one of the most important effector molecules targeted by ATM [6]. The ATR kinase activates a pathway principally induced by UV damage which involves Chk1 kinase [7–9]. The most important targets of both Chk1 and Chk2 are members of the Cdc25 phosphatase family. These molecules are normally required for the activation of cyclin-dependent kinase. Once phosphorylated, Cdc25a is functionally inhibited and prevents the activity of cyclin-dependent kinase-cyclin complexes involved in the transition G1/S, and in the progression through S and G2/M, triggering G1, S and G2/M checkpoints [10–12]. In addition, p38*α*/*β*-dependent activation of MK2 works in a different cell cycle checkpoint kinase pathway activated in response to UV as well as to currently used chemotherapeutic drugs. MK2 functions are critical especially in cells and tumors losing p53 [13–18]. A relatively DDR slow response is the ATM/ATR-dependent p53 phosphorylation, with consequent transcriptional activation of genes involved in cell cycle arrest (i.e., page 21) [3]. At this point, if DNA lesions are adequately repaired, cells can proceed to proliferate again and to inactivate DNA damage checkpoints; otherwise they undergo to apoptosis [19]. DNA damage can be repaired by different mechanisms. The base excision repair (BER) and the nucleotide excision repair (NER) complexes remove damaged and modified nucleotides. In particular, NER works principally on helix-distorting and transcription-blocking lesions (i.e., UV-induced pyrimidine dimers), whereas BER removes single nucleotides modified by methylation, alkylation, deamination, or oxidation [20]. The mismatch repair machinery (MMR) operates through MSH2 and MLH1, which form heterodimers with MSH3 or MSH6 and MLH3, PMS2 or PMS1, respectively. These complexes are involved in mismatch/insertion-deletion loops recognition and in the excision-repair reaction [21, 22]. MMR machinery removes incorrectly incorporated nucleotides during DNA synthesis or replication errors in DNA repeats causing microsatellite instability [23]. DNA double strand breaks (DSBs) are frequent events in eukaryotic cells: they are induced by physiological mechanisms in early lymphocytes or by pathological activity attributable to ROS, ionizing radiation or erroneous nuclear enzyme functions. DSBs are repaired by non-homologous end joining (NHEJ) in all phases of the cell cycle, thus representing the major pathway in G1 or homologous recombination (HR) in the S or G2 phases of the cell cycle [24, 25]. NHEJ involves the binding of the heterodimeric Ku protein to double-stranded DNA ends, the recruitment of the DNA-dependent protein kinase catalytic subunit (DNA-PKcs), and DNA-PK kinase activation [24]. The DNA-PK complex on the DNA works by recruiting a complex containing DNA ligase IV, XRCC4, and XLF/Cernunnos protein, with consequent rejoining [24]. HR is a complex process involving the generation of 3′ single-stranded DNA overhang first bound by RPA. The displacement of the complex by Rad51 follows; then a homologous sequence is engaged to repair the lesion [25].

Tumor cells frequently acquire the ability to overcome the DDR network: in fact, most cancers show malfunctions in the DDR, such as resistance to genotoxic agents and ionizing radiations or abnormal cell cycle progression after DNA damage [26]. The tumor suppressor p53 is one of the most important proteins activated after DNA damage, and p53 gene mutations are described in almost every type of cancer, at rates comprised between 38% and 50% in ovarian, colorectal, head and neck, and lung cancers [27]. Similarly, some hereditary cancer predisposition syndromes such as ataxia telangiectasia, Li-Fraumeni, and Nijmegen syndromes, breast and ovarian cancer or colon cancer (HNPCC) predisposition, and Xeroderma pigmentosum show lesions in genes involved in the DDR machinery (ATM, p53, NBS1, BRCA-1/2, MMR genes, and NER genes) [23]. In response to DNA damage, the mRNA expression patterns go through significant modifications [28, 29] which can be due to RNA PolII hyperphosphorylation with consequent prevention of the formation of preinitiation complexes at sites of the promoter [30]. In this context, post-transcriptional regulation of mRNAs mediated by microRNAs (miRNAs) plays also a fundamental role.

## 3. Biogenesis and Functions of MicroRNAs

MicroRNAs (miRNAs) are a class of short noncoding RNAs, usually 18–25 nucleotides in length, mediating important cellular functions such as proliferation, apoptosis, differentiation, and cell signaling [31–33]. MiRNAs regulate the translation of specific mRNA by their imperfect or perfect pairing at the level of the 3′UTR [34]. It is estimated that in the human genome approximately 30% of genes are targeted by miRNAs [35]. MiRNAs genes are transcribed in two different ways: long primary miRNAs (pri-miRNAs) are synthesized from intergenic miRNAs by RNA polymerase II, whereas intronic or esonic miRNAs are transcribed in association with their host genes from a common promoter [36]. After cleavage of pri-miRNAs in the nucleus by the microprocessor Drosha-DGCR8, precursor pre-miRNAs (approximately 70 nucleotides in length) are obtained. Pre-miRNAs are then exported from the nucleus to the cytoplasm by the RanGTP-binding nuclear transporter exportin-5. In the cytoplasm, the endoribonuclease Dicer cleaves pri-miRNAs, generating a duplex about 25 bases in length which consists of a functional miRNA and a passenger strand. The association between Argonaute (Ago) proteins and mature miRNAs generates RISC (RNA-induced silencing complex) which leads to post-transcriptional gene regulation, consisting of mRNA degradation or translation inhibition [37]. Due to their role in the regulation of fundamental cellular functions, miRNAs are involved in several pathological processes and, in particular, in carcinogenesis [38].

## 4. MicroRNAs in DNA Damage/Repair Mechanisms and Cancer

### 4.1. MicroRNAs in ATM and ATR Pathways

ATM gene has been shown to be downregulated by miR-421 in neuroblastoma and HeLa cells [39]. MiR-421 targeted the ATM 3′UTR, playing a role in modulating cell cycle checkpoints and cellular radiosensitivity. Ectopic expression of miR-421 led to S-phase cell cycle checkpoint changes and radiosensitivity increase and was able to generate an ataxia telangiectasia-like phenotype. The inhibition of the interaction between miR-421 and ATM 3′UTR reverted the effects due to miR-421 overexpression. Furthermore, overexpression of N-myc, a gene frequently amplified in neuroblastoma, was able to induce miR-421 expression with consequent ATM downregulation, suggesting a correlation between N-myc-mediated oncogenesis and the network involving miRNAs and molecules working in the DNA damage/repair machinery. Ng et al. [40] demonstrated that miR-100 was highly expressed by M059J glioblastoma DNA-PK-deficient cells. DNA-PK is a molecule playing a role in repairing IR-induced double strand breaks. MiR-100 was responsible for the low expression of ATM detected, besides DNA-PK deficiency, in the same cells. MiR-100 can target the 3′UTR of ATM gene and its knocking-down induced ATM expression. Yan et al. [41] identified miR-101 as a molecule able to sensitize cancer cells to IR by targeting the 3′UTR of DNA-PKcs and ATM transcripts. The authors demonstrated that the miR-101 overexpression could be used for rendering tumor cells more sensitive to radiations in “*in vitro*” and “*in vivo*” models. MiR-18a was able to affect the DNA damage response mechanisms through ATM downregulation [42]. MiR-18a was over-expressed in breast cancer cell lines and tumors and its ectopic expression downregulated ATM by direct interaction with the 3′UTR of the gene. ATM siRNA and miR-18a overexpression caused reduction of homologous recombination and DNA repair in breast cancer cells, making them more sensitive to ionizing radiations. Conversely, the inhibition of miR-18a led to increase of homologous recombination and DNA repair efficiency, thus reducing cellular radiosensitivity. In addition, the authors showed that miR-18a overexpressing cells displayed significantly reduced phosphorylation and nuclear foci formation of H2AX and 53BP1, which are downstream ATM substrates. Accordingly, Wu et al. [43] demonstrated that miR-18a was over-expressed in colorectal cancers and was responsible for the diminishment of DNA damage repair by suppressing ATM expression. A recent research pointed-out the role of ATM itself in regulating miRNAs biogenesis. Zhang et al. [44] reported that about 25% of miRNAs were upregulated upon DNA damage in an ATM-dependent manner and that ATM loss abolished miRNAs induction. ATM directly phosphorylated KSPR (KH-type splicing regulatory protein), a component of the Drosha and Dicer miRNA processing complexes. This induced increased interaction between KSPR and pri-miRNAs and enhanced pri-miRNAs processing activity by Drosha microprocessors and stimulation of miRNA maturation. Since the expression of KSRP was found to be suppressed in several types of human cancer (breast, esophagus, kidney, liver, and testis), KSRP-induced miRNA biogenesis could be inhibited in these cancers, with consequent deregulation at the level of pathways and molecules' functions. This work provided further evidence about the role of ATM in tumorigenesis. A very early step in the response of mammalian cells to DNA double-strand breaks is the phosphorylation of histone H2AX by ATM [45]. Wang et al. [46] used the human osteosarcoma cell line U2OS to develop a screening assay based on the evaluation of radiation-induced *γ*H2AX (phosphorylated histone H2AX) foci formation. Several miRNAs able to inhibit *γ*H2AX foci formation were identified. MiR-138 specifically targeted the H2AX 3′UTR, reducing its expression and inducing chromosomal instability after DNA damage. MiR-138 overexpression inhibited homologous recombination and increased sensitivity to DNA damaging agents. Conversely, the expression of histone H2AX in miR-138 overexpressing cells resulted in the attenuation of these effects. In this context, miR-138 expression could potentially improve the efficacy of DNA damaging agents against tumor cells. Tsai et al. [47] examined the role of the carcinogen areca nut extract in inducing miRNAs modulation in human oral fibroblasts. Areca nut-induced miR-23a overexpression was correlated with an increase of the DNA damage marker *γ*H2AX and a reduction of DSB repair in “*in vivo*” plasmid assay. The Fanconi anemia susceptibility gene FANCG, playing a role in DSB repair process, was predicted to be a target of miR-23a. FANCG expression was reduced by areca nut-induced miR-23a overexpression, generating inhibition of DSB repair. MiR-23a overexpression was also described in oral cancers from areca-nut chewing patients. Chang et al. [48] showed that miR-3928 was induced by ionizing radiations in HeLa cells and targeted the endoribonuclease Dicer. MiR-3928 overexpression promoted ATR activation and Chk1 phosphorylation. MiR-3928 overexpression was also able to downregulate several miRNAs, including miR-185, 300, and 663. Additionally, Wang et al. [49] demonstrated that miR-185, whose expression is reduced after ionizing radiation exposure in renal cell carcinoma (RCC), targeted ATR. MiR-185 expression sensitized RCC cells to X-rays both “*in vivo”* and “*in vitro”* and enhanced radiation-induced apoptosis as well as inhibition of proliferation by repressing ATR pathway. Therefore, miR-185 could be potentially used to radiosensitize cancer cells.

### 4.2. MicroRNAs in p53 Pathway

Chang et al. [50] analyzed the p53 wild-type HCT116 colon cancer cell line in comparison to an isogenic cell line with both p53 alleles inactivated by homologous recombination. After treatment with the genotoxic agent adriamycin, able to induce p53 and its downstream targets, 474 human miRNAs were analyzed. Seven miRNAs (miR-23a, miR-26a, miR-34a, miR-30c, miR-103, miR-107, and miR-182) were upregulated in p53^wt^ cells. Among them, miR-34a showed the highest expression change after adryamicin administration, resulted transcriptionally regulated by p53 and was able to induce apoptosis. Accordingly, miR-34a was decreased in pancreatic cancer cells, which frequently show p53 loss-of-function. MiR-34a induction produced a strong reprogramming of expression of genes acting in regulating cell-cycle progression, proliferation, apoptosis, angiogenesis, and DNA repair (upregulation of RAD51AP1, CHEK1, and MDC1). Accordingly, another study [51] demonstrated that miR-34 family was directly regulated by p53 in cell lines and mouse tissues in response to genotoxic drugs and ionizing radiations. By using antisense oligonucleotides and the mouse ES cell line, where miR-34a was genetically inactivated, BCL2 was found to be regulated by miR-34. MiR-34a variant was also described to be downregulated in neuroblastoma lacking the 1p36 allelic region encompassing the miR-34a gene [52]. It has been described that 1p36 genomic loss is an event common to different types of cancers [52], indicating that miR-34a activity could play a pivotal role in tumor suppression. Furthermore, miR-34b/c was dramatically reduced in non small cell lung cancers (43%), and the restoration of the expression caused growth inhibition of NSCLCs cells [51]. MiR-34b/c was also able to enhance cellular radiosensitivity of malignant pleural mesothelioma cells by promoting radiation-induced apoptosis. As shown by *γ*H2AX foci assay, DBS repair was delayed in miR-34b/c transfected cells. MiR-34b/c inhibited the expression of cyclin-D1, CDK4/6, and BCL2 and enhanced cleaved caspase-3 and poly (ADP-ribose) polymerase cPARP levels after irradiation [53]. Mir-34c was described as being upregulated in a study by Josson et al. [54], where LNCaP and C4-2 prostate cancer cell lines were used to evaluate global miRNA expression after radiation treatment. Several miRNAs were found to be differentially expressed, and significant changes were observed in miR-521 (downregulated) and miR-34c (upregulated) expression. MiR-34c upregulation was attributable to p53 regulation. Regarding miR-521, the authors demonstrated that miR-521 mimics sensitized prostate cells to radiation, whereas its ectopic inhibition led to radiation resistance. This effect was ascribed to the miR-521 predicted targeted proteins Cockayne syndrome A (CSA) and MnSOD (manganese superoxide dismutase), involved in DNA repair and in oxidative processes, respectively. Radiation treatment inversely correlated the expression levels of CSA and MnSOD and miR-521. Due to its role, miR-521 was described to be a potential target for the enhancement of radiation therapy on prostate cancer cells. Shin et al. [55] developed a model where the NSCLC A549 cell line was treated with different doses of ionizing radiations. MiRNA profiles were analyzed and several differentially expressed miRNAs involved in many cellular functions such as apoptosis, cell cycle control, and DNA damage/repair were identified in response to different radiation doses. Among the latter, miR-34a (DNA damage/repair predicted target genes PCBP4, POLD1), miR-34b* (target genes ATR, ERCC5, and RFC5), miR-192 (target genes CDK7, ERCC3, ERCC4, and XPA), miR-215 (target genes CDK7, ERCC3, ERCC4, TDG, and XPA), miR-376a (target genes ATR, MNAT1, and NEK11) were found over-expressed, whereas miR-106a (target genes MSH3, GTF2H3, and POLH), miR-548c-3p (target genes CDK7, MSH2, PCNA, PMS2), miR-760 (target gene BRCA-1), miR-16-2* (target genes EXO1, MSH2, PCNA, POLD4, and SETX), miR-139-3p (target genes POLA1, POLE), miR-345 (target genes LIG1, POLD3, POLL, RFC1, and RPA1), miR-516a-5p (target genes ASF1A, BRCA1, GTF2H4, and MBD4) were described as being downregulated. A study [56] was focused on the analysis of time-course changes of miRNA expression induced by the genotoxic agent N-ethyl-N-nitrosourea (ENU) in mice. MiR-34 family members (miR-34a, miR-34b, miR-34c) were upregulated during the ENU treatment and were described as potential biomarkers for genotoxic exposure. Saleh et al. [57] analyzed the mechanisms causing downregulation of let-7a/b, in response to agents able to induce oxidative and radiation damage. They demonstrated that this effect was dependent “*in vitro*” on p53 and ATM, since it was not observed in the p53^−/−^ HCT116 colon cancer cell line and in ATM^−/−^ human fibroblasts. The decreased expression of let-7 was triggered by p53 binding on a region upstream of the let-7 gene following radiation exposure. Radiation-sensitive tissues also showed “*in vivo*” radiation-induced let-7a/b downregulation, and this effect was not observed in p53 knock-out mice, indicating that let-7 regulation occurred by p53-mediated repression. Accordingly, exogenous expression of let-7a/b enhanced radiation-induced cytotoxicity in HCT116 p53^+/+^, but not in those p53^−/−^. MicroRNAs play also a role in modulating the expression of p53 regulatory factors. Among these, the wild-type p53-induced phosphatase 1 (Wip1), a member of serine/threonine phosphatases which is frequently over-expressed in several tumors, plays a critical role in DNA damage signaling by dephosphorylating DNA-damage responsive proteins in the ATM/ATR-p53 pathway [58]. A study by Zhang et al. [59] showed that miR-16 was induced after DNA damage and targeted Wip1. Overexpression of miR-16 or inhibition of Wip1 was able to suppress mouse mammary tumor stem cells growth and to enhance the response to doxorubicin in MCF-7 cells. Expression levels of mdm2, a known regulator of p53, are also affected by miRNAs. Suh et al. [60] analyzed glioblastoma multiforme cells and demonstrated that miR-25 and miR-32 were repressed by a p53-dependent negative regulation of their transcriptional factors E2F and myc. At the same time, miR-25 and miR-32 were able to target mdm2, generating p53 accumulation and an autoregulatory circuitry. Dar et al. [61] investigated the role of miR-18b, whose expression was found to be reduced in melanoma samples through methylation. The authors identified mdm2 as a target of miR-18b and demonstrated that its overexpression in melanoma cells resulted in mdm2 downregulation, with consequent p53 upregulation and p53 pathway reactivation. Amir et al. [62] demonstrated that miR-125b targeted p14^arf^ as well as p53, Puma, and Bak1 in prostate cancer cells [63]. MiR-125b inhibited also the interaction between p14^arf^ and mdm2, affecting the p14^arf^/mdm2/p53 pathway activity and apoptosis induction. On the contrary, anti-miR-125b treatment restored p14^arf^ expression, apoptosis induction, and decreased mdm2 levels. Avasarala et al. [64] showed that miR-29b acts as a tumor suppressor in NSCLC by regulating mdm2 expression. In addition, miR-661 was described as a negative regulator of mdm2 and mdm4 in several cell lines from melanoma, lung, breast, and ovarian cancer. High miR-661 expression was correlated with good prognosis in breast cancer, mostly expressing wild type p53 [65].

### 4.3. MicroRNAs in MMR Machinery

Lanza et al. [66] analyzed colon cancer samples characterized by microsatellite stability (MSS) or instability (MSI-H). The study was focused on the analysis of mRNAs and microRNAs expression. Eight miRNAs correctly distinguished MSI-H from MSS samples. Among these, several members of the miR-17-92 family (miR-17-5p, miR-20, miR-25, miR-92-1, miR-92-2, and miR-93-1, miR-106a) were upregulated in MSS with respect to MSI-H colon cancers. The miR-17-92 cluster was described as being upregulated in NSCLC [67] and its ectopic overexpression enhanced lung cancer cells growth, suggesting that these molecules may act as oncogenes and might have a role in more aggressive clinical behavior of MSS with respect to MSI-H tumors. Sarver et al. [68] analyzed miRNA profiles in defective MMR (dMMR, showing microsatellite instability and/or MLH1 protein absence) and proficient MMR (pMMR, with absence of microsatellite instability and presence of normal MLH1) colorectal cancers in comparison to adjacent normal colon samples. Six miRNAs were differentially expressed: miR-31 and miR-625 were over-expressed in dMMR, whereas miR-552, miR-592, miR-181c, and miR-196b were described as being decreased in pMMR samples. Valeri et al. [69] analyzed miRNAs involved in mismatch repair mechanisms and found that the overexpression of miR-155 caused downregulation of the mismatch repair proteins MLH1, MSH2, and MSH6, with consequent mutator phenotype and MSI in colorectal cancer cell lines. MiR-155 acted by targeting the 3′UTR regions. MiR-155 overexpression was inversely correlated to MLH1 and MSH2 expression in colorectal cancer samples and some MSI cancers with undetectable cause of MMR machinery inactivation alike showed miR-115 overexpression. Valeri et al. [70] demonstrated that miR-21 targets the core MMR recognition protein complex, MSH2 and MSH6, in colon tumor cells. Colorectal tumors overexpressing miR-21 showed reduced levels of MSH2 protein and cells overexpressing miR-21 displayed reduced 5-fluorouracil-induced G2/M damage arrest and apoptosis. Xenograft models confirmed that miR-21 overexpression was able to reduce 5-fluorouracil efficacy. Therefore, miR-21 expression could be considered a marker of therapy efficacy in colorectal cancer. MiR-21 function was also studied by Yu et al. [71], who analyzed breast cancer cells. They identified miR-21 as a miRNA regulated by TGF-beta, a cytokine with tumor suppressor activity in normal cells, but able to promote malignancy in cancer if abused [72]. The authors demonstrated that TGF-beta-induced miR-21 caused downregulation of MSH2 expression by 3′UTR targeting. An inverse correlation between TGF-beta and MSH2 expression was also described in primary breast tumors, confirming the relationship between the cytokine and miR-21 activity. Recently, Zhang et al. [73] used a system based on A549 cells to demonstrate that cisplatin treatment downregulated miR-21 expression, with consequent MSH2 increase and A549 cells growth inhibition. Oberg et al. [74] analyzed samples from normal colonic mucosa, tubulovillous adenoma, and proficient MMR, deficient MMR sporadic, and hereditary colon cancers. Six miRNAs (miR-31, miR-135b, miR-9, miR-1, miR-99a, and miR-137) were differentially expressed in adenoma versus normal samples (fold changes more than 4). All of them, except miR-99a, showed comparable expression differences in normal versus carcinoma comparisons, providing evidence that these changes are common early events both in pMMR and dMMR cancers. MiR-31, miR-552, miR-592, and miR-224 were differentially expressed in proficient versus deficient MMR cancers, providing evidence of miRNAs involved in distinguishing pMMR and dMMR. MiR-99a was over-expressed (fold change less than 4) in pMMR and dMMR versus adenoma comparisons. MiR-99 family expression was also correlated with radiation sensitivity and was able to target the SWI/SNF chromatin remodeling factor SNF2H/SMARCA5, a component of the ACF1 complex [75]. Mir-99a and miR-100 were involved in the reduction of the localization of BRCA-1 at the DNA damaged sites. Expression of miR-99 family in cells diminished the level of both HR and NHEJ efficiency and miR-99a induction by radiations prevented the SNF2H increase and reduced the recruitment of BRCA-1 at the damaged sites after a second dose of radiations. This decreased the repair efficiency upon multiple doses of radiations. Since the radiation therapy is usually performed by administering fractionated doses of radiations, miR-99 expression could play a role in affecting the efficacy of this treatment.

### 4.4. MicroRNAs in NER Machinery

Some works have shown that hypoxia can also promote genetic instability by affecting the DNA repair capability of cancer cells, due to transcriptional downregulation of MLH1, MSH2, BRCA-1, and RAD51 observed in hypoxic cells [76–79]. Crosby et al. [80] used HeLa, MCF-7, and mouse embryonic fibroblast to analyze the role of miRNAs in DNA repair under hypoxic stress. MiR-210 and miR-373 were upregulated in hypoxic cells in a hypoxia-inducible factor-1 alpha- (HIF-1*α*-) dependent manner. Forced expression of miR-210 was able to suppress levels of RAD52, a key factor in homology-dependent repair, whereas miR-373 overexpression caused downregulation of RAD23B, a component of the XPC/RAD23B complex involved in the NER machinery, as well as RAD52. MiR-210 and miR-373 interacted with the 3′UTR of RAD52 and RAD23B, respectively. MiR-210 and miR-373 inhibition by antisense strategy can partially reverse the hypoxia-induced RAD52 and RAD23B downregulation. Friboulet et al. [81] analyzed the role of excision repair cross-complementation group 1 (ERCC1), a NER pathway protein involved in recognition and removal of DNA platinum adducts and in repair of stalled DNA replication forks, in NSCLCs from a cohort of 91 patients. ERCC1-positive tumors showed lower rate of genomic lesions with respect to ERCC1 negative. MiR-375 was found to be reduced in ERCC1-positive cancers. MiR-375 was previously described as being downregulated in gastric cancer and hepatocellular carcinoma (HCC) and was able to inhibit HCC proliferation [82, 83]. Genes involved in DNA repair, such as TP53, USP1, APEX1, TYMS, MLH3, PARP4, NTHL1, ERCC3, and XRCC6BP1, were predicted to be targeted by miR-375. Therefore, miR-375 downregulation could determine a proliferative advantage and also an increased DDR phenotype in ERCC1-positive tumors. The HBV-expressing HepG2.2.15 cell line, with impaired NER activity, showed upregulation of miR-192 [84]. ERCC3 and ERCC4, two proteins involved in NER pathway, were downregulated by miR-192, with consequent impairing of NER machinery. The role of polymorphisms at the level of miRNA binding sites of 28 NER genes was correlated with colorectal cancer risk, by analyzing cohorts of about 1,000 cancers and 1,500 healthy controls [85]. Two polymorphisms, rs7356 in RPA2 and rs4596 in GTF2H1, were associated with colorectal cancer risk, indicating that alterations in miRNA target binding sites may play a role in tumorigenesis.

### 4.5. MicroRNAs in Regulation of Genes Involved in Different DNA Repair Pathways and Mechanisms

Chaudhry et al. [86] studied differentially expressed miRNAs after IR treatment in glioblastoma, which often show resistance to radiation therapy. Glioblastoma cells (M059 K and M509J) with normal or deficient DNA-dependent protein kinase (DNA-PK) activity and resistance or sensitivity to ionizing radiations were used. The let-7 family, known to be a RAS regulator, which is upregulated in cells showing normal DNA-PK activity, whereas the same family was downregulated in cells with DNA-PK deficient activity. MiR-17-3p, miR-17-5p, miR-19a, miR-19b, miR-142-3p, and miR-142-5p were upregulated in cells with normal and deficient DNA-PK activity. MiR-15a, miR-16, miR-143, miR-155, and miR-21 showed upregulation in cells with normal DNA-PK activity and varied in a time-dependent manner in DNA-PK deficient cells. Among these miRNAs, miR-155 was associated with c-myc overexpression, whereas miR-15a and miR-16 were described as being able to target BCL2, whose gene product acts in protecting cells from IR-induced apoptosis and confers resistance to DNA damage [87]. MiR-21 was already described to participate in a miRNA targeting a network including p53, TGF-beta, and mitochondrial apoptosis tumor suppressor genes in glioblastoma [88]. Moskwa et al. [89] analyzed breast cancer cells to demonstrate that miR-182 was responsible for the downregulation of BRCA-1 expression, whose decreased expression is observed in 30–65% of sporadic basal-like tumors. MiR-182 levels decreased after IR in a p53-independent manner, being detectable both in p53-proficient (MCF-7, HMEC) and deficient (K562, HL60) cell lines. MiR-182 antagonism led to increase of BRCA-1 expression, protecting cells from IR-induced cell death, whereas miR-182 overexpression reduced BRCA-1 expression with consequent defects in HR-mediated repair. Mir-182 overexpressing tumor cells were hypersensitive to inhibitors of poly (ADP-ribose) polymerase 1 (PARP1); on the contrary, miR-182 antagonism led to enhanced BRCA-1 levels and induced resistance to PARP1 inhibitors. This research focuses on the importance of miR-182 overexpression in affecting the response to PARP1 inhibitors. In a study by Krishnan et al. [90], miR-182-5p was frequently upregulated in human breast cancers and was able to target a network of genes involved in DNA repair, identified by synthetic biotinylated miRNA to pull down endogenous miR-182-5p targets in HEK293T cells. Rui et al. [91] used a docetaxel-resistant NSCLC (SPC-A1/docetaxel) and its parental cell line to perform miRNA differential expression analysis. Three upregulated (miR-192, miR-424, and miR-98) and 3 downregulated miRNAs (miR-200b, miR-212, and miR-194) were identified in docetaxel-resistant cells. Moreover, approximately 90 predicted target genes involved in DNA damage/repair (among them, XPA, RAD1, XPC, TP53, BRCA-1, SIRT1, MSH2, RAD50, and ATM) were described. Teo et al. [92] analyzed in bladder and breast cancer patients common single nucleotide polymorphisms (SNPs) potentially involved in miRNA binding sites in the 3′UTR of 20 genes involved in DNA repair. They found SNPs (PARP1 rs8679 T>C and RAD51 rs7180135 A>G) associated with increased bladder and breast cancer risk and with improved cancer specific survival following radiation therapy in bladder cancer. Predicted miRNAs involved in this process were miR-145, miR-105, miR-630, and miR-302a (regulation of PARP1) and miR-197 (regulation of RAD51). Yan et al. [93] analyzed the role of RAD21, an essential molecule for chromosome segregation as well as high-fidelity DNA repair by homologous recombination with BRCA-1/2, in affecting the prognosis of BRCA-2, BRCAX, and BRCA-1 familial breast cancers. High RAD21 expression was associated with genomic instability and miR-299-5p, a microRNA already involved in breast cancer and in oral squamous cell carcinoma, was predicted to be a RAD21 regulator. Wang et al. [94] demonstrated that the expression of RAD51 and REV1 polymerase, involved in the resistance to DNA interstrand crosslinking agents, was downregulated by miR-96 in several types of cancer cells. MiR-96 was able to directly target the coding region of RAD51 and the 3′UTR of REV1. Overexpression of miR-96 was a negative regulator of RAD51 foci formation, induced decrease of the efficiency of homologous recombination and enhancement of the sensitivity to the AZD2281 PARP inhibitor “*in vitro*” and to cisplatin both “*in vivo*” and “*in vivo*”, suggesting that miR-96 mimics could be used to enhance cancer chemosensitivity. Zheng et al. [95] used an artificial approach to analyze the effect of an engineered miRNA (amiR) able to target in a perfect complementary way the 3′UTR of XRCC2, a fundamental homologous recombination factor, and XRCC4, an essential nonhomologous end joining factor, in cancer cells along with a siRNA. XRCC2 and XRCC4 showed higher expression in tumor tissues and cells with respect to non cancer tissues/cells. The artificial amiR efficiently inhibited the expression of XRCC2 and XRCC4 genes, sensitizing human tumor cells to radiation-induced death. This effect was further enhanced by combining amiR to siRNA to target both the noncoding and coding regions of XRCC2 and XRCC4. Hegre et al. [96] demonstrated that miR-16, miR-34c, and miR-199a downregulated the expression of the BER complex protein nuclear uracil-DNA-glycosylase UNG2 in a 3′UTR targeting dependent process.

## 5. Clinical Potentials of MiRNA-Based Cancer Therapy

Cancer treatment is usually based on chemo- and/or radiotherapy. Unfortunately, many tumors exhibit radio- or chemoresistance, with consequent efficacy reduction of these treatments. During last decade, small molecules or protein drugs, such as monoclonal antibodies, specifically directed against molecules with dysregulated activity, have been generated and used in association with conventional therapies [97–100]. Due to their functions, microRNAs are considered target molecules and potentially effective therapeutic options for cancer. Some studies have shown that miRNA mimics may be administered without side effects in non-human primates and mice, reducing tumor growth [101–103]. Therefore, depending on their role in promoting or suppressing cancer, miRNA expression could be either selectively inhibited (i.e., anti-miR oligonucleotides, miRNA sponges) or endogenously/exogenously restored. In this context, microRNA delivery by nanoparticles potentially able to specifically target tumors, for example, through the use of RNA aptamers or chemical ligands, is starting to play an emerging role in medicine and clinical practice [104]. MiRNAs involved in the regulation of DNA damage/repair mechanisms can be considered markers to predict the response to radiotherapy and utilized hereafter to define personalized treatments [105] (Table 1). In this regard, expression levels of miRNAs could be evaluated in serum and/or tumor specimens to predict radiosensitivity and optimal radiation dose, in order to make the treatment more effective and to limit both side effects and normal tissue injury. In addition, expression and activity of miRNAs able to affect the response to chemo- or radiotherapy could be specifically modified and modulated to enhance the expected therapeutic effects. The use of artificial miRNAs with sequences able to target genes already known to have important roles in DNA damage/repair mechanisms could be of great impact in regulating mechanisms able to render cancer cells more sensitive to DNA damaging agents.

## 6. Conclusions

MiRNAs play a pivotal role in various biological and pathological processes driving to cancer initiation, progression, and metastasis formation. The analysis of microRNA-modulated gene regulation in the DDR and its involvement in cancer pathogenesis and progression will help to understand and define the impact of these small molecules in DNA damage/repair as well as chemo- and radioresistance mechanisms. This knowledge will expand the characterization of molecules and networks involved in pathways activated upon DNA damage and the subsequent alterations at the level of fundamental processes such as cell cycle control and apoptosis. The identification of miRNA-modulated genes and the effects of miRNAs deregulated functions will make it possible to acquire information about the prognosis, the chemo- or radioresistance, and then the response to therapeutic treatments in cancer. In conclusion, functional characterization of the network including proteins able to repress or induce miRNAs as well as miRNAs and targets will provide significant information about prognosis and therapy of cancer.

## Figures and Tables

**Table 1:** MiRNAs involved in DNA damage/repair and 3′-UTR targeted genes.

MiRNA	3′UTR targeted genes	Reference
miR-421	ATM	Hu et al., 2010 [39]
miR-100	ATM	Ng et al., 2010 [40]
miR-101	ATM, DNA-PK	Yan et al., 2010 [41]
miR-18a	ATM	Song et al., 2011 [42]; Wu et al. 2013 [43]
miR-138	H2AX	Wang et al., 2011 [46]
miR-210	RAD52	Crosby et al., 2009 [80]
miR-3248	Dicer	Chang et al., 2012 [48]
miR-185	ATR	Wang et al., 2013 [49]
miR-16	Wip1	Zhang et al., 2010 [59]
miR-25, miR-32	MDM2	Suh et al., 2012 [60]
miR-18b	MDM2	Dar et al., 2013 [61]
miR-661	MDM2	Hoffman et al., 2013 [65]
miR-34a	BCL2	Bommer et al., 2007 [51]
miR-155	MLH1, MSH2, and MSH6	Valeri et al., 2010 [70]
miR-21	MSH2	Yu et al., 2010 [71]
miR-192	ERCC3, ERCC4	Xie et al., 2011 [84]
miR-373	RAD23B	Crosby et al., 2009 [80]
miR-96	REV1	Wang et al., 2012 [94]
miR-16, miR-34c, and miR-199a	UNG2	Hegre et al., 2013 [96]
